# Reproductive health of women with and without disabilities in South India, the SIDE study (South India Disability Evidence) study: a case control study

**DOI:** 10.1186/s12905-014-0146-1

**Published:** 2014-11-30

**Authors:** Gudlavalleti Venkata S Murthy, Neena John, Jayanthi Sagar, South India Disability Evidence Study Group

**Affiliations:** South Asia Centre for Disability Inclusive Development and Research, Indian Institute of Public Health, Public Health Foundation of India, ANV Arcade, 1 Amar Cooperative Society, Kavuri Hills, Madhapur, Hyderabad, 500033 India

**Keywords:** Disabled persons, Health care disparities, India, Pregnancy, Public health

## Abstract

**Background:**

Evidence shows that women with disability have adverse pregnancy outcomes compared to those without a disability. There is a lack of published data on reproductive health of women with disability in India till date. The objective of the South India Disability Evidence (SIDE) Study was to compare reproductive health parameters including pregnancy experience, health access during pregnancy and type of delivery among women with disability compared to women with no disability.

**Methods:**

The study was conducted in one district each in two States (Andhra Pradesh and Karnataka) in 2012. A case-control design was used to identify appropriate age and sex-matched controls for women with disability identified through a population-based survey. Trained key informants first listed women with disabilities who were then examined by a medical team to confirm the diagnosis. Trained research investigators administered questionnaire schedules to both groups of women to collect information on reproductive health and outcomes of any pregnancy experienced in the past two years.

**Results:**

A total of 247 women with disability and 324 age-matched controls aged 15-45 years were recruited for the study. 87% of the women with disability had a physical disability. The mean age of women with disability was 29.86 against 29.71 years among women without a disability. A significantly lower proportion of women with disability experienced pregnancy (36.8%) compared to women without a disability (*X*^2^ –16.02 P <0.001). The odds ratio for suffering from diabetes among women with disability compared to women without a disability was 19.3(95% CI: 1.2- 313.9), while it was 9.5 (95% CI: 2.2-40.8) for depression. A higher proportion of women without a disability (7.7%) compared to women with a disability (5.3%) reported a successful pregnancy in the past two years. There were no statistically significant differences between women with and without a disability with regard to utilization of antenatal care and pregnancy outcomes.

**Conclusions:**

The study provides evidence on some reproductive health parameters of women with disability in India for the first time ever. The findings will help in formulating policy and to develop specific interventions to improve pregnancy outcomes for women with disability in India.

## Background

Globally, people with disability are recognized as one of the most marginalized and socially excluded groups [1]. Even among people with disability, women with disability fare worse as they are the most disadvantaged in the social ladder in many countries including India [2–7]. The Millennium Development Goals 4 and 5 which relate to gender equality and maternal health can only be achieved if women with disability attain equal access to maternal health services as women without a disability [8]. The reproductive health needs of women with disability have not received much attention in the past, most probably due to the misguided notion that women with disability are not sexually active and are not desirous of bearing children [9]. Times have changed and more women with disability are experiencing pregnancy than ever before [10]. In fact women with disability perceive a sense of normality of their existence because of pregnancy, as it affirms their capacity to enjoy motherhood [11].

Evidence from qualitative studies shows that the health care needs of women with disabilities have not been adequately met in many parts of the world [12–15]. These studies have highlighted barriers to accessing reproductive health care services for women with disability and the lack of preparedness of the health care system to cater to their needs. The commonest barriers that people with disability face in accessing health services are physical access barriers, communication barriers or lack of awareness from the providers about issues concerning people with disabilities [16,17]. Studies in low income countries document that barriers are also created due to prevalent cultural norms in a country [17].

There is a complete lack of published literature in peer reviewed journals on the reproductive health status of women with disabilities in India. The objective of the present study was to compare some of the reproductive health care experiences of women with disability with women with no disability. The reproductive health experiences included experiencing pregnancy, no. of living children, no. of miscarriages, health care access in pregnancy and type of delivery. We believe that this will help us to bridge the evidence gap to enable need-based appropriate reproductive health services for women with disability in India.

## Methods

The present study is part of a larger study undertaken in the State of Andhra Pradesh, called the SIDE Study (South India Disability Evidence Study) [18]. The study was conducted over a ten month period in 2012, to identify women with disability and age-matched neighborhood controls to ascertain their reproductive health issues, pregnancy outcomes and health service utilization.

### Study area

One district each with poor social indicators from Andhra Pradesh (Medak district) and Karnataka (Bidar district) were included in the study. One administrative division of comparable population was randomly identified in each district (Sangareddy - Medak; Bidar taluka - Bidar).

### Sample size

The sample size was estimated using a power of 80%, significance level of 0.05, 95% confidence intervals and a difference of 10% in pregnancy outcomes among women with and women without a disability. It was decided to include 1.5 controls for every case (woman with a disability). The estimated sample size was 222 women with disability (cases) and 333 women without a disability (controls).

### Data collection

Cases were defined as married women with disability aged 15-45 years, who were resident in the study area continuously for at least the last six months, prior to the data collection. They were recruited from the community. Controls were identified from the same neighbourhood as the cases but not from the same household. They were married women aged 15-45 years with no disability and resident for at least the prior six months in the same community. Cases were initially identified by key informants and then confirmed by the trained field investigators using a reference manual and corroborated with the findings on the disability certificates issued to all people with disabilities in India or disability pension records if available. The ‘Persons with disabilities’ Act in India includes visual impairment, hearing impairment, locomotor impairment/orthopedically handicapped, mental illness/handicap, including persons with multiple disabilities/impairments. People with any of the above impairments are examined by a medical board at the district level which certifies the extent of the disability before issuing a ‘Disability Certificate’. The controls were identified by the field investigators immediately after a case was identified in the community. All participants were drawn from the rural areas.

The study used a two-stage process to identify the cases. In the first stage, key informants (KIs) were recruited from the study area and trained to identify people with disability. The KIs were trained using a specially designed and pretested flip book with pictorial depictions of the different impairments on identification of persons with disability, based on visible impairments/abnormalities and a brief history. The training was conducted in a village within the study district. All KI were transported to the training site. The duration of training was one day. Approximately 20 KIs were trained per selected block (smallest administrative area) (approximately 1.5-2 persons per selected village) and their participation was voluntary, without material reward throughout the process. Each KI covered a population of between 2000-3000 over a period of 4 to 6 weeks, going house to house. At the end of 6 weeks the KI provided the list of people with disability to trained field investigators. The field investigators visited each of the persons listed by the KI. They reconfirmed the findings of the KI and simultaneously identified age- matched neighbourhood controls, without any disability. All the identified individuals were then administered a questionnaire schedule to elicit responses regarding reproductive health care issues in addition to recording basic demographic data. The questionnaires were translated into the local languages (Telugu and Kannada) and were pretested before use.

All field investigators and KI’s were people with disabilities.

In the second stage, a team of a medically trained physician and a therapist visited all listed individuals (people with disability and controls) at home to confirm the diagnosis and examined them in detail for their underlying impairment and for re-ascertaining the information collected by the field investigators.

If a woman with disability could not respond to the questionnaire schedule due to severe impairment, a responsible adult member of the household was asked to respond to the questionnaire (proxy respondent).

### Disabilities included in the study

Since the study used KI for the initial listing of people with disabilities, it was possible to include only those impairments which were visible to the external eye or could be picked up through a short history. The following impairments were included in the study:

- Physical impairments: Club foot, cleft lip, cleft palate, cerebral palsy, Down’s syndrome, microcephaly, phocomelia, amputated limb, burns, muscular dystrophy/atrophy, leprosy, elephantiasis, post-polio residual paralysis, congenital limb deficiencies, rickets and spinal cord injuries
- Visual Impairment: Bilateral severe visual impairment or blindness
- Hearing Impairment: Bilateral severe/profound hearing impairment
- Intellectual impairment.

People with disability were defined as those who suffered from one or more of the impairments as listed as these impairments are responsible for disability due to activity limitation and effect on social participation.

### Ethics

The ethical approval for the study was obtained from the Institutional Ethical Committee at Indian Institute of Public Health- Hyderabad.

### Consent

Written informed consent was obtained from all the study participants. Where the participant could not provide consent due to a disability, consent was sought from a responsible adult member of the household.

### Statistical analysis

The data base was developed in MS ACCESS and STATA 12.0 was used for data analysis. The chi-square test was used for associations. Multivariate analysis was performed using Mantel and Haenszel stratification technique in Stata 12.0 to determine the adjusted odds ratio for associated variables. The Mantel and Haenszel stratification technique allows to adjust for confounding. Age and number of live births were considered as confounding variables. In computing odds ratios, history of diabetes and depression were also adjusted for confounding when the other variable’s odds ratio was being computed. In the Mantel and Haenszel stratification technique the exposure outcome is stratified into multiple tables, each corresponding to a fixed level of the confounding variable. In the second step, the (crude) odds ratio is calculated for each table. The adjusted odds ratio is then produced by taking a weighted average (weights being proportional to the cell frequencies of each table) of the odds ratio.

### Services

All participants were provided referral linkages to tertiary care centres for treatment wherever required. Transportation was organized for the people with disabilities to reach the tertiary centres. Treatment provided included surgery and provision of assistive devices.

## Results

A total of 571 women aged 15-45 years were included in the study. The ratio of women with disability to women without a disability was 1:1.3 (Cases: 247 women with a disability; Controls: 324 women without a disability).

Physical impairment was the commonest impairment (74.5%) among women with disability enrolled in the study (Table 1). Women with other impairments were also represented in the study. They were much smaller numbers as the prevalence of these impairments is relatively lower compared to physical impairment in India.

**Table 1:** **Distribution of impairments among women with disability**

**Impairment**	**% No. (N** = **247)**
Blindness/Visual Impairment	2.8% (7)
Intellectual Impairment	4.4% (11)
Moderate/Severe Hearing Impairment	5.7% (14)
Generalized convulsive disorders	12.5% (31)
Physical Impairments	74.5% (184)
Distribution of Physical Impairments	N = 184
Post-polio residual paralysis	44.6% (82)
Limb deformities including phocomelia	23.4% (43)
Amputated Limb	9.2% (17)
Joint Deformity	6.5% (12)
Cerebral Palsy	2.7% (5)
Burns	2.7% (5)
Club foot	1.6% (3)
Other Physical Impairments	9.2% (17)

Women with and without a disability were similar in their demographic characteristics (Table 2). The mean age of women with disability was 29.86 against 29.71 years among women without a disability (*X*^2^-0.727; p = 0.394). The proportion that was illiterate was similar between the two groups. However a significantly higher proportion of women without a disability had been educated to graduation or beyond, compared to none among women with a disability (*X*^2^- 5.3; p = 0.02).

**Table 2:** **Demographic characteristics of women with disability and age-matched controls**

**Parameters**	**Women with disability % (No.)**	**Women without disability % (No.)**
Aged 15-45 years (Reproductive Age Group)	43.26% (247)	56.74% (324)
Mean Age (years)	29.86 [SD: 8.8]	29.71 [SD:8.4]
Literacy Status		
Illiterate	59.1 (146)	58.0% (188)
	*X* ^2^ - 0.03; p = 0.86	
Completed Graduation	0.0% (0)	2.8% (9)
	*X* ^2^ – 5.3; p =0.02	
Work as daily wage labor	81.7% (125)	76.1% (169)
	*X* ^2^ – 0.08; p =0.77	

Reproductive health experiences differed significantly between the two groups. A significantly lower proportion of women with disability experienced pregnancy (36.8%) compared to women without a disability (53.7%) (*X*^2^-16.02; p <0.001) (Table 3). Despite this, women with disability had more living children compared to women without a disability. There was a significant difference between the proportion of women with disability reporting diabetes (9.7%) compared to women without a disability(0.6%) (*X*^2^-26.7; p <0.001). Similar significant differences on univariate analysis was observed with depression where the difference between women with disability (14.6%) and women without a disability (1.5%) were significantly different (*X*^2^- 35.71; p <0.001). Multivariate analysis showed that the adjusted odds ratio for diabetes was 19.3 (95% CI: 1.2- 313.9) for women with disability compared to women without a disability while the adjusted odds ratio for women with disability compared to women without a disability for depression was 9.5 (95% CI: 2.2-40.8) (Table 4).

**Table 3:** **Reproductive health of women with and without a disability (univariate analysis)**

**Parameters**	**Women with disability % (N-247)**		**Women without disability % (N-324)**	
	**% (No.)**		**% (No.)**	
Ever Pregnant	36.8% (91)		53.7% (174)	
	*X* ^2^ –16.02 P <0.001			
Mothers reporting any live birth	36.03% (89)		53.1% (172)	
Mean live children	2.04	SD: 0.99	1.95	SD:0.88
History of Past Miscarriages	11.7% (29)		14.2% (46)	
	*X* ^2^ –; p = 0.31			
History of Diabetes	9.7% (24)		0.6% (2)	
	*X* ^2^ –26.70; p <0.001;			
History of Hypertension	0.9% (2)		1.4% (4)	
	*X* ^2^ –0.283; p =0.594			
History of Depression	14.6% (36)		1.5% (5)	
	*X* ^2^ –35.71; p <0.001;

**Table 4:** **Systemic problems reported by women with disability compared to women without a disability (multivariate analysis)**

**Parameters**	Adjusted odds ratio*	**95% confidence interval**
History of Diabetes		
Women without disability	1.0	
Women with Disability	19.3	1.2 – 313.9
History of Depression		
Women without disability	1.0	
Women with disability	9.5	2.2 – 40.8

*Adjusted for age, history of live births, depression and diabetes.

Women who had delivered a live born during the past two years were administered additional questions regarding the last pregnancy. A higher proportion of women without a disability (7.7%) compared to women with a disability (5.3%) reported a successful pregnancy in the past two years (Table 5). Delivery at hospitals and delivery through surgical Cesarean section were commoner among women without a disability but these differences were not statistically significant. Women with disability reported less attention during their pregnancy by health personnel compared to peers without a disability, but these differences were again not statistically significant. Comorbidities like convulsions and depression were reported to be significantly higher among women with a disability, though there was no difference in relation to diabetes and hypertension between the two groups (Table 5).

**Table 5:** **Maternal health issues of women with and without disability**

**Parameters**	**Women with disability (N-247)**	**Women without disability (N-324)**
	**% (No.)**	**% (No.)**
History of pregnancy in last 2 years	5.3% (13)	7.7% (25)
	*X* ^2^ –1.36; p = 0.24; Not significant	
	**N = ** **13**	**N = ** **25**
Baby delivered at hospital in the pregnancy in last 2 years	69.2% (9)	72.0% (18)
	*X* ^2^- 0.04; p =0.86; Not significant	
Baby delivered by Caesarean section in the pregnancy in last 2 years	38.5% (5)	40.0% (10)
	*X* ^2^- 0.07; p =0.80; Not significant	
Received adequate advice from health worker/ doctor during the pregnancy in last 2 years	30.8% (4)	56.0% (14)
	*X* ^2^-1.29; p = 0.26; Not significant	
Report not being examined regularly during the pregnancy in last 2 years	30.8% (4)	8.0% (2)
	*X* ^2^-1.84; p = 0.15; Not signficant	
Reported complications during delivery in the pregnancy in the last 2 years	23.1% (3)	20.0% (5)
	*X* ^2^- 0.04; p = 0.84: Not significant	
H/0 Diabetes among women reporting pregnancy in past 2 years	7.67% (1)	0.0% (0)
H/0 Hypertension among women reporting pregnancy in past 2 years	0.0% (0)	4.2% (1)
H/0 convulsions among women reporting pregnancy in past 2 years	30.8% (4)	0.0% (0)
	*X* ^2^- 8.5973; p =0.003; Significant	
H/0 depression among women reporting pregnancy in past 2 years	46.1% (6)	0.0% (0)
	*X* ^2^- 13.7019; p < 0.001; Significant	

## Discussion

India ratified the UNCRPD in 2007 and has been providing a legal framework for implementation of services for people with disabilities through the adoption of the Persons with Disability (PWD) Act, 1995 [19]. The PWD Act set out the processes for people with disability to access employment and rehabilitation opportunities and affirmative action to be initiated to provide the needed services [20]. The PWD Act talks about equal opportunities and non-discrimination but does not cover any affirmative action to improve the health status or health care access of persons with disabilities. In the absence of such directives, the health system has not invested in special programs focusing on health for people with disability. We observed that reproductive health parameters like pregnancy experience, antenatal care, type of delivery, number of living children etc. were comparable between women with and without a disability in India. We believe that affirmative action could provide excellence in all aspects of reproductive health for women with disability.

This study is the first attempt to unravel and document the differences between women with and without disability with regard to their reproductive health experiences in India.

Nearly eight out of every ten women with disability recruited for this study had some form of functional physical disability and therefore the findings reflect the concerns of women with physical impairments much more than the other impairments.

The overall demographic characteristics of women with and without a disability were similar except that none of the women with a disability had studied to college level. This is an observation that has been reported even from economically developed countries [21].

Women with a disability reported significantly lower pregnancy experience compared to women without a disability. Similar findings have been reported earlier [22]. At the same time, we observed that women with disability had more live children compared to women without a disability. This could be because contraceptive use may be lower amongst women with disability compared to women without a disability. We did not specifically look at contraceptive use in the study and so this may be speculative.

It has been documented from previous studies that adults with lifelong disabilities are more likely to have poorer health because of chronic diseases compared to those without a disability [23]. Review of data from the US showed that adults with lifelong disabilities had increased odds of coronary heart disease, cancer, diabetes, obesity and hypertension, showing thereby that disability is likely to result in poor health [23]. Studies have shown that women with disabilities have a higher risk of depression compared to men with disability [24]. It has also been reported that diabetes is associated with physical disability [25] as well as with intellectual disability [26,27]. The higher risk of non-communicable diseases like hypertension and diabetes is likely to be related to the relative lack of physical activity among people with disability, resulting in higher rates of obesity among people with disability compared to the general population [28]. It has been hypothesized that in addition, there is an impairment of nutritional status among people with disability due to a quantitative and qualitative inadequacy of diet which could be the harbinger of co-morbidities among them [29].

It has been reported that the pregnancy outcomes and complications among women with disability are significantly higher compared to the general population. Evidence from some populations has shown that women with disability had significantly higher rates of premature delivery and low birth weight among the newborn [30]. The rate of pregnancy-related complications as well as urinary tract infections was significantly higher among women with disability [30]. At the same time, just as we observed, women with disability were reported to have been accessing antenatal services as much if not more compared to women without a disability [8,31]. This is a positive sign but more proactive steps are needed to improve the capacity of the health work force to deal with the needs of women with disability as some studies have highlighted the lack of confidence and skill of the health workforce in dealing with women with disability [11,32].

In conclusion we find that pregnancy and reproductive health are as important for women with disabilities compared to the general population, if not more. This is because each pregnancy is precious to women with disability as it gives them a sense of being perceived as a normal human being as any other woman. The health system in India is able to reach out to women with disability and there are no significant differences on many parameters of reproductive health. Developing appropriate communication material and dealing with the special needs of these women will empower them significantly and bring about a change in societal attitudes that women with disability have the same biological and reproductive needs as any other woman in society. This realization is critical to the success of universal improvement in maternal health and reduction of maternal mortality and morbidity in a country like India, where women with disability encounter high levels of stigma.

### Limitations of the study

The study had a few limitations. Women who had delivered a child in the last two years were asked questions on the last pregnancy; this had possibility of recall bias. The other limitation of the study was that sample size was small especially of women who experienced pregnancy in the last two years, which would result in low power to detect smaller differences between experiences of women with and without disability.

## Conclusions

The study demonstrates that women with disability have a significantly lower pregnancy rate compared to women without a disability. Women with disability have significantly higher risk of co-morbidities like diabetes and depression. Contrary to what has been reported from many countries, parameters related to ante-natal and natal care were similar for women with and without a disability. There is an urgent need to collect more data on pregnancy experiences and reproductive health concerns of women with disability using much larger samples.

## References

[1] Lang R, Kett M, Groce N, Trani JF (2011). Implementing the United Nations convention on the rights of persons with disabilities: principles, implications, practice and limitations. Eur J Disability Res.

[2] Mitra S, Posarac A, Vick B (2013). Disability and poverty in the developing countries: a multidimensional study. World Dev.

[3] Piotrowski K, Snell L (2007). Health needs of women with disabilities across the lifespan. J Obstet Gynecol Neonatal Nursing.

[4] Smith DL (2008). Disparities in health care access for women with disabilities in the United States from the 2006 national health interview survey. Disability Health J.

[5] Chevarley FM, Thierry JM, Gill CJ, Ryerson AB, Nosek MA (2006). Health, preventive health care, and health care access among women with disabilities in the 1994-1995 national health interview survey, supplement on disability. Womens Health Issues.

[6] Nicolau SM, Schraiber LB, Ayres JR (2013). Women with disabilities and their double vulnerability: contributions for setting up comprehensive health care practices. Cien Saude Colet.

[7] Ferri BA, Gregg N (1998). Women with disabilities: missing voices. Wom Stud Int Forum.

[8] Trani JF, Browne J, Kett M, Bah O, Morlai T, Bailey N, Groce N (2011). Access to health care, reproductive health and disability: a large-scale survey in Sierra Leone. Soc Sci Med.

[9] Groce N, Trasi R (2004). Rape of individuals with disability: aIDS and the folk belief of “virgin cleansing”. Lancet.

[10] Blackford K, Richardson H, Grieve S (2000). Prenatal education for mothers with disabilities. J Advanced Nursing.

[11] Walsh-Gallagher D, Sinclair M, McConkey R (2012). The ambiguity of disabled women’s experiences of pregnancy, childbirth and motherhood: a phenomenological understanding. Midwifery.

[12] Lipson JG, Rogers JG (2000). Pregnancy, birth and disability: women’s health care experiences. Health Care Women Int.

[13] Prilleltensky O (2003). A ramp to motherhood: the experiences of mothers with physical disabilities. Sex Disabil.

[14] Nosek MA, Hughes RB, Howland CA, Young ME, Mullen PD, Shelton ML (2004). The meaning of health for women with physical disabilities: a qualitative analysis. Fam Community Health.

[15] Thomas C, Curtis P (1997). Having a baby: some disabled women’s reproductive experiences. Midwifery.

[16] Iezzoni LI (2009). Public health goals for persons with disabilities: looking ahead to 2020. Disabil Health.

[17] Bremer K, Cockburn L, Ruth A (2010). Reproductive health experiences among women with physical disabilities in the Northwest region of Cameroon. Int J Obstet Gynaecol.

[18] Gudlavalleti MV, Neena J, Komal A, Sagar J, Kamalakannan S, Ramachandra SS (2014). Access to health care and employment status of people with disabilities in South India, the SIDE (South India Disability Evidence) study. BMC Public Health.

[19] Choudhary LN, Shikha D (2013). Indian legal system and mental health. Indian J Psychiatry.

[20] The Persons with disabilities (Equal opportunities, protection of rights and full participation) Act 1995: http://www.thenationaltrust.co.in/nt/images/stories/pwdact1995.pdf. Accessed 12^th^ June 2014.

[21] Iezzoni LI, Yu J, Wint AJ, Smeltzer SC, Ecker JL (2013). Prevalence of current pregnancy among US women with and without chronic physical disabilities. Med Care.

[22] Iezzoni LI, Yu J, Wint AJ, Smeltzer SC, Ecker JL (2014). General health, health conditions, and current pregnancy among US women with and without chronic physical disabilities. Disabil Health J.

[23] Dixon-Ibarra A, Horner-Johnson W (2014). Disability status as an antecedent to chronic conditions: National Health Interview Survey, 2006-2012. Prev Chronic Dis.

[24] Niemeier JP (2008). Unique aspects of women’s emotional responses to disability. Disabil Rehabil.

[25] Reichard A, Stolzle H, Sellla AC, Shireman TI (2012). Quality of diabetes care for adults with physical disabilities in Kansas. Disabil Health J.

[26] Taggart L, Coates V, Truesdale-Kennedy M (2013). Management and quality indicators of diabetes mellitus in people with intellectual disabilities. J Intellect Disabil Res.

[27] McVilly K, McGillivray J, Curtis A, Lehmann J, Morrish L, Speight J: **Diabetes in people with intellectual disability: a systematic review of prevalence, incidence and impact.** *Diabet Med* 2014, May 13 [epub ahead of print]. 10.1111/dme.12494 24824086

[28] Mudge S, Kayes NM, Stavric VA, Channon AS, Kersten P, McPherson KM (2013). Living well with disability: needs, values and competing factors. Int J Behav Nutr Phys Act.

[29] Bertoli S, Battezzati A, Merati G, Margonato V, Maggioni M, Testolin G, Veicsteinas A (2006). Nutritional status and dietary patterns in disabled people. Nutr Metab Cardiovasc Dis.

[30] Morton C, Le JT, Shahbandar L, Hammond C, Murphy EA, Kirschner KL (2013). Pregnancy outcomes of women with physical disabilities: a matched cohort study. PM R.

[31] Redshaw M, Malouf R, Gao H, Gray R (2013). Women with disability: the experience of maternity care during pregnancy, labour and birth and the postnatal period. BMC Pregnancy Childbirth.

[32] Morrison J, Basnet M, Budhathoki B, Adhikari D, Tumbahangphe K, Manandhar D, Costello A, Groce N: **Disabled women’s maternal and newborn health care in rural Nepal: a qualitative study.** *Midwifery* 2014, S0266–S6138 [Epub ahead of print]. 10.1016/j.midw.2014.03.012 PMC4217148 24768318

